# Oxygen saturation for the prediction of acute mountain sickness

**DOI:** 10.1177/20552076241310086

**Published:** 2024-12-19

**Authors:** Johannes Burtscher, Martin Faulhaber, Martin Kopp, Martin Burtscher

**Affiliations:** 1 27255 Department of Sport Science, University of Innsbruck, Innsbruck, Austria

**Keywords:** high altitude, oxygen saturation, pulse oximetry, acute mountain sickness, prediction

## Abstract

We read with interest the paper on “Smartwatch measurement of blood oxygen saturation for predicting acute mountain sickness: Diagnostic accuracy and reliability” recently published by Zeng and colleagues in Digital Health. This study demonstrated good reliability and high precision for measuring peripheral oxygen saturation (SpO_2_) using Huawei smartwatches during a 1-wk high altitude exposure. In addition, SpO_2_ values recorded during the ascent to high altitude were predictive for the development of acute mountain sickness (AMS). Although these findings are valuable and in accordance with previous studies, some additional clarifications would be helpful to better understand the use of SpO_2_ and its clinical relevance as an AMS predictor.

Dear Editor,

We read with great interest the article on “Smartwatch measurement of blood oxygen saturation for predicting acute mountain sickness: Diagnostic accuracy and reliability” recently published by Zeng and colleagues in Digital Health.^1^ This study demonstrated good reliability and high precision in the measurement of peripheral oxygen saturation (SpO_2_) using Huawei smartwatches during a 1-wk high altitude exposure. In addition, SpO_2_ values taken during the ascent to high altitude were predictive for the development of acute mountain sickness (AMS).^1^ Although these findings are valuable and in accordance with previous studies,^2^ some additional clarifications would be helpful for better understanding of the use and the clinical relevance of SpO_2_ as an AMS predictor.

First, the ascent profile during the first 3 days differed significantly between groups 1 and 2, with group 1 ascending to 5200 m and group 2 to 2780 m on the first day and staying at that altitude for the following 2 days.^1^ Unfortunately, the authors do not report AMS data for day 3 when SpO_2_ values are most different between groups. However, this would be highly important because data probably demonstrate the favorable effects of staying 2 days at the same (lower) altitude and its beneficial consequences on the subsequent ascent. In addition, it would be desirable to know figures on AMS incidence based on the AMS categorization (Lake Louise AMS Scores, LLS) mentioned in the method section (mild: 3–5 points and moderate: 6–9 points), instead of showing data for LLS 3–4 points and ≥5 points). Furthermore, it might have been helpful to inform the reader about some methodological problems discussed with the LLS-self-rating instrument.^3^

Second, the authors refer to the low sub-sample of females but do not report SpO_2_ values and the AMS incidence of female participants. This, however, would also be desirable (despite the low numbers) because females may be more vulnerable to AMS^4^ and thus more benefit from the lower ascent profile of group 2.

Third, the authors state that the increase in hematocrit during the first days contributes to the attenuated oxygen transport capacity. However, this may not be true when the authors refer to the capacity of oxygen delivery. Oxygen delivery (DO_2_) equals cardiac output (CO) times arterial oxygen content (CaO_2_) and CaO_2_ equals hemoglobin concentration (Hb) times arterial oxygen saturation (SaO_2_). Because hematocrit increases with the elevation of Hb, this would improve DO_2_, provided CO and SaO_2_ remain unchanged. Thus, it would be of interest to know changes in hematocrit or Hb levels (if available). Somewhat lower SaO_2_ levels may be compensated by higher hematocrit values, which should be kept in mind when interpreting CaO_2_ only by SaO_2_ values.
